# Proof of Concept in Artificial-Intelligence-Based Wearable Gait Monitoring for Parkinson’s Disease Management Optimization

**DOI:** 10.3390/bios12040189

**Published:** 2022-03-23

**Authors:** Robert Radu Ileșan, Claudia-Georgiana Cordoș, Laura-Ioana Mihăilă, Radu Fleșar, Ana-Sorina Popescu, Lăcrămioara Perju-Dumbravă, Paul Faragó

**Affiliations:** 1 Department of Neurology and Pediatric Neurology, Faculty of Medicine, University of Medicine and Pharmacy “Iuliu Hatieganu” Cluj-Napoca, 400012 Cluj-Napoca, Romania; robert.ilesan@usb.ch (R.R.I.); popescu.ana.sorina@elearn.umfcluj.ro (A.-S.P.); lperjud@elearn.umfcluj.ro (L.P.-D.); 2 Clinic of Oral and Cranio-Maxillofacial Surgery, University Hospital Basel, CH-4031 Basel, Switzerland; 3 Bases of Electronics Department, Faculty of Electronics, Telecommunications and Information Technology, Technical University of Cluj-Napoca, 400114 Cluj-Napoca, Romania; claudia.cordos@bel.utcluj.ro (C.-G.C.); laura.mihaila@bel.utcluj.ro (L.-I.M.); 4 Computer Science, Faculty of Mathematics and Computer Science, West University of Timișoara, 300223 Timișoara, Romania; radu.flesar02@e-uvt.ro

**Keywords:** artificial intelligence, sensors, convolutional neural networks, Parkinson’s disease, biomedical monitoring, accelerometer, pressure sensor, disease management, electromyography, correlation

## Abstract

Parkinson’s disease (PD) is the second most common progressive neurodegenerative disorder, affecting 6.2 million patients and causing disability and decreased quality of life. The research is oriented nowadays toward artificial intelligence (AI)-based wearables for early diagnosis and long-term PD monitoring. Our primary objective is the monitoring and assessment of gait in PD patients. We propose a wearable physiograph for qualitative and quantitative gait assessment, which performs bilateral tracking of the foot biomechanics and unilateral tracking of arm balance. Gait patterns are assessed by means of correlation. The surface plot of a correlation coefficient matrix, generated from the recorded signals, is classified using convolutional neural networks into physiological or PD-specific gait. The novelty is given by the proposed AI-based decisional support procedure for gait assessment. A proof of concept of the proposed physiograph is validated in a clinical environment on five patients and five healthy controls, proving to be a feasible solution for ubiquitous gait monitoring and assessment in PD. PD management demonstrates the complexity of the human body. A platform empowering multidisciplinary, AI-evidence-based decision support assessments for optimal dosing between drug and non-drug therapy could lay the foundation for affordable precision medicine.

## 1. Introduction

More than 200 years ago, in 1817, Dr. James Parkinson published a scientific work entitled “The Essay on Shaking Palsy” [1] and with it the foundation of the disease that bears his name. After two centuries, we still struggle to understand and treat neurodegenerative diseases, such as Parkinson’s disease (PD), which are growing exponentially, especially in industrialized regions, and given that no one is immune to them, specialists are concerned about “the Parkinson pandemic” [2].

In 2017, we started the PDxOne project, intending to optimize the management of PD by offering sustainable automated or semiautomated solutions.

### 1.1. Considerations on Parkinson’s Disease

Parkinson’s disease (PD) is a common progressive neurodegenerative disorder that can cause significant disability and decreased quality of life [3]. PD is today more present than ever, being the second most common neurodegenerative disease after Alzheimer’s disease and affecting 0.3% of the population [4]. It is estimated that there are 6.2 million people diagnosed with PD disease worldwide, and the disease caused the death of 117,000 people in 2015 alone [5]. The American Parkinson Disease Association estimates that there are already 1 million people with PD living in the U.S. alone and over ten million worldwide, giving some researchers the evidence of a Parkinson pandemic [2]. While studies are divided on the prevalence by gender, the affinity of the pathology for the aging tissue, particularly neural tissue, is much clearer. Statistically, PD occurs in people over 60 years old, affecting 1% of this age group and increasing to 4% for people over 80 [4]. Unfortunately, PD can also occur in younger people under 50, known as Young Onset Parkinson’s disease (YOPD). Studies show that 5–10% of patients diagnosed with PD are between 20 and 50 years old [6]. It is becoming even more complex with the literature showing cases of patients diagnosed with PD who were younger than 20—even some rare cases with patients under ten years of age, and the first symptoms appeared as early as two years of age [7]. This form is known as the juvenile form of PD and was first described in 1875. It has a substantial genetic component that can be diagnosed today with genetic testing [7].

In 2014, Mary Ann Thenganalt et al. performed a systematic review of articles cited in PubMed between 1980 and 2013. They concluded that PD could be divided into several subtypes, the most representative being tremor-dominant and postural instability gait difficulty form (PIGD) [8]. The clinical presentation of PD can take the form of many symptoms, from which the easiest to notice are the motor impairments [9]. These symptoms are caused by the degeneration of the dopaminergic neurons located in the substantia nigra from the ventral midbrain [10]. According to the Movement Disorder Society (MDS), the clinical diagnosis of PD is based on the presence of bradykinesia, along with either rest tremor or rigidity [11].

Motor impairment in PD has long been the focus of researchers, with significant advances being made in diagnostic accuracy, implementation, improvement of more accurate assessment scales, and better management of therapeutic strategies [12]. Unfortunately, at the moment of the diagnosis, a significant number of neurons that produce dopamine are already dysfunctional [6]. During the 15–20 years before the onset of motor symptoms, the patients experience a phase called “prodromal PD”, during which the neurodegeneration starts and progresses [13]. It has been proved that olfaction impairment, constipation, depression, rapid eye movement (REM) and sleep behavior disorder (RBD) can be present in the prodromal period of PD [14].

Although the focus of research has been on motor symptoms, clinical studies have shown that non-motor symptoms in PD, such as depression, pain, psychosis and sleep disturbances, should be regarded as equally important when analyzed using quality-of-life questionnaires as well as economic and health indicators [12]. Therefore, we are dealing with a pathology that is increasingly present in our lives, that will double its number globally by 2030 [15], and that will have a significant socio-economic impact. Socially, PD patients become isolated, stigmatized [16], and even discriminated, with severe implications for the course of the disease and the clinical picture. To diagnose PD in a patient means to put a verdict that irreversibly alters their lifestyle and that of their families. Once diagnosed, the patient will also undergo a lifelong treatment, which aims not to cure but to improve or stagnate the symptoms’ evolution. Studies, therefore, show a mortality rate double that of the healthy population, which increases and presents a more aggressive clinical picture in patients with YOPD or juvenile PD [17]. Therefore, the aspect of early onset raises many questions about our current ability to understand the pathophysiology of PD and treatment errors that may result in individual and global socio-economic consequences.

Indeed, medical assistance for patients with PD is a major drain on the healthcare budget. This financial amount is given by the complexity of PD, which affects the patient on several levels at once. To understand the real-life implications that affect every one of us and why we should spend the necessary resources, a quantification of socio-economic indicators is necessary. One figure we could start with is the financial effort that countries in Europe make to treat patients with PD, i.e., almost EUR 14 billion per year [18]. Interestingly, this figure also represents the amount the USA is spending per year treating PD patients, namely USD 14.4 billion [19]. The average annual cost per patient for PD in Germany is EUR 20,095 [20]. On average, direct costs represent 65.5% and indirect costs 34.5% [21]. Of the total direct costs (EUR 13,158), EUR 3526 is spent on medication, which is also the largest expenditure, and EUR 3789 is spent annually on hospitalization and home care costs [21]. Costs for home care by the family amount to 20% of the direct costs [21]. The same study shows a directly proportional relation between costs and disease progression, i.e., EUR 18,660 annual costs for stage 1–2, increase to EUR 31,660 annual costs for stage 2–5 (according to Hoehn and Yahr) [21]. Annual costs for PD differ quite a lot between European countries. While in Russia, EUR 5240 is spent per patient per year [21], in England, the annual costs for a patient with advanced stage PD (3–5 according to Hoehn and Yahr) can reach EUR 72,277 [22]. Attention is required, especially regarding the increase in treatment costs if patients are taken out of their environment, out of their home, and moved to a nursing home. In this case, studies show a 500% increase in costs for PD treatment [23]. All these costs are strictly related to PD, but a patient may also have other associated pathologies aggravated by PD and vice versa. Moreover, the financial impact on society is difficult to quantify because it should be taken on a patient-by-patient basis.

A staggering EUR 798 billion is spent annually at the European level to treat brain diseases, according to a study carried out in 2010 [19]. This amount is almost four times higher than Romania’s GDP. It shows the importance of continuous research to provide a sustainable medical system for Europe and beyond in a growing and aging population that wants to maintain its standard of living in old age.

These figures motivate the ongoing research on wearables for the early detection of PD-specific symptomatology and prediagnosis of PD in incipient stages, as well as long-term monitoring of the disease in a ubiquitous healthcare environment which provides intelligent decision support algorithms for assessment and patient-specific treatment plans in PD.

### 1.2. Related Work—Wearables in PD Monitoring

For exemplification, Boroojerdi et al. report on the employment of the NIMBLE wearable biosensor patches, composed of an accelerometer and an electromyography (EMG) sensor, for motor evaluation in PD [24]. As for another example, Jauhiainen et al. report on the employment of a Movesense sensor and a Forciot insole to observe walking patterns in PD [25]. Phan et al. report on the use of BioKin devices to assess daily tasks: pointing, pouring, walking, and walking around a chair [26]. Lonini et al. report on employing BioStamplRC flexible wearable sensors, consisting of a tri-axial accelerometer and gyroscope, to record motion data [27].

Continuous long-term monitoring of motor symptoms in PD using inertial sensors is described by Borzì et al., aiming for the identification of bradykinesia and FOG [28], or by Powers et al., aiming for the identification of tremors and dyskinesia [29]. The employment of built-in smartphone sensors, with dedicated smartphone applications, is described by Heijmans et al. in [30] or by Motolese et al. in [31], for the remote monitoring of the PD patients during daily activities.

### 1.3. Related Work—AI-Based Decisional Support in PD Assessment

Some noteworthy examples regarding AI-based decisional support are presented as follows: Lonini et al. report on the employment of Random Forest classifiers to identify bradykinesia and tremor [27]. Random Forest for classification in PD was also studied by Aich et al. in [32], along with support vector machine, K-Nearest Neighbor, and Naïve Bayes. PD-specific symptomatology detection and classification using convolutional neural networks (CNN) was reported by Taewoong et al., who assessed daily activities based on 3D acceleration and angular velocity data measured with a Microsoft Band 2 [33]. Further employment of CNN was reported by Lonini et al. for the identification of bradykinesia and tremor [27], and by Steinmetzer et al. for the arm oscillation monitored with Mbientlab portable Motion Rectangle sensor bracelets under a Timed Up and Go (TUG) test scenario [34].

### 1.4. This Work

The PDxOne research project desires to develop and use the latest technologies to collect and, above all, interpret medical data. With a world population reaching 8 billion people and with today’s medical requirements and demands, these tasks simply cannot be hand-operated anymore. Given the sheer medical data volume that must be collected every day, the request for economical and sustainable solutions forces healthcare systems to embrace a worldwide digitalized implementation.

Thus, our work is placed in the context of today’s demand for ubiquitous monitoring and intelligent decisional support in healthcare. We target to develop, at the end of our project, a small-size wearable and portable monitoring system for patients diagnosed with PD, aiming for long-term quantitative and qualitative assessment of the PD symptomatology in a continuous fashion, and intended for AI-based decisional support in the discrimination of the pathology and formulation of dedicated treatment plans, which constitutes a novelty in the field.

This article targets the monitoring and assessment of gait in patients diagnosed with PD. Gait disorders are a hallmark of the condition and are associated with a loss of independence and an increased risk of falls. Disturbances of the gait, even if hardly noticeable, are described from the earliest stages of the disease [35] and include shuffling gate, shortened stride length, reduced overall velocity, and increased stance phase (up to doubling), along with reduced or absent arm swing, reduced trunk rotation, and decreased amplitude of motion in the hips, knees, and ankles [3]. In advanced stages, gait disorders often become increasingly complex, including motor blocks, festination, and imbalance [36].

Multiple studies have also been conducted for the early detection of motor deficient behaviors to apply proper therapeutic interventions, which are proved to slow down the motor dysfunction and maintain functional independency (in patients with preserved cognitive function) [3,37,38,39]. Symptoms such as dyskinesia, which is induced by therapy and manifests as involuntary movement of any body parts, appear in advanced stages of PD [9]. The symptomatic therapy for the classic motor features is usually satisfactory, but antiparkinsonian therapy that does not induce motor complications is still needed [40].

The solution proposed for ubiquitous gait monitoring and AI-based decisional support in gait assessment is envisioned in the shape of a wearable physiograph. The proposed physiograph performs bilateral tracking of the foot biomechanics assessed by means of plantar pressure distribution and lower-limb EMG, in correlation to upper limb balance, which is evaluated by means of arm balance magnitude of acceleration (MA) and variation of acceleration (VA). The recorded signals are transmitted over a Bluetooth radio link to a mobile device, e.g., smartphone or tablet. They are uploaded and stored into an online database and made available for future access, either in real-time or offline, for processing and interpretation.

The proposed physiograph enables both qualitative and quantitative assessment of gait. As such, we perform gait evaluation based on biomechanical parameters, expressed in terms of arm balance, heel strike, and lift-off, and temporal parameters, expressed in terms of cadence, single support, double support, single support to double support ratio, and stride time variability. Next, we evaluate the physiological interdependencies involved during the gait cycle by applying the cross-correlation function to each recorded signal pair. We illustrate that PD-specific gait is identifiable based on the evaluated gait assessment parameters following the evaluation results. Consequently, the biomechanical and temporal parameters and the cross-correlation results are applicable as inputs to an expert system for identifying and discriminating PD-specific gait pathology.

The novelty of the proposed physiograph consists of the underlying AI-based decisional support procedure for gait assessment. We generate a correlation coefficient matrix from the gait monitoring signals to visually represent the gait pattern. Gait assessment using the biomechanical and temporal parameters and the cross-correlation function is contained in the correlation coefficient matrix. Then, we apply the surface plot of the correlation coefficient matrix to a convolutional neural network (CNN) for gait classification.

A proof of concept of the proposed physiograph with AI-based decisional support is validated in the clinical environment on a group of ten subjects consisting of five PD patients and five healthy controls. As such, the proposed solution provides a feasible method for AI-based support for gait monitoring and assessment in a ubiquitous healthcare environment.

## 2. Materials and Methods

This paper proposes a wearable miniature physiograph with AI-based decisional support for gait monitoring and assessment in PD. Gait evaluation is performed in accordance with the Unified Parkinson’s Disease Rating Scale (UPDRS)—motor subscale, and the Movement Disorder Society UPDRS (MDS-UPDRS) [41,42].

The proof of concept of the proposed wearable gait monitoring physiograph was tested extensively in the laboratory and validated indoors in the clinical environment, with a study group consisting of five patients diagnosed with PD and five healthy controls. The PD group includes three males and two females. The healthy control group includes four males and one female. The healthy controls do not have any previously diagnosed neurodegenerative disorder or podiatric condition.

All procedures performed in this study involving human participants were following the ethical standards of the institutional and/or national research committee. Informed consent was obtained from all individual participants involved in the study.

### 2.1. The Proposed Physiograph for Gait Monitroing in PD

**Figure 1:**
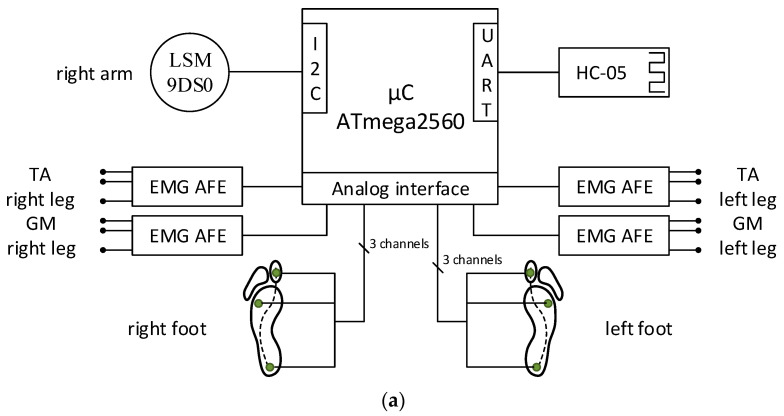

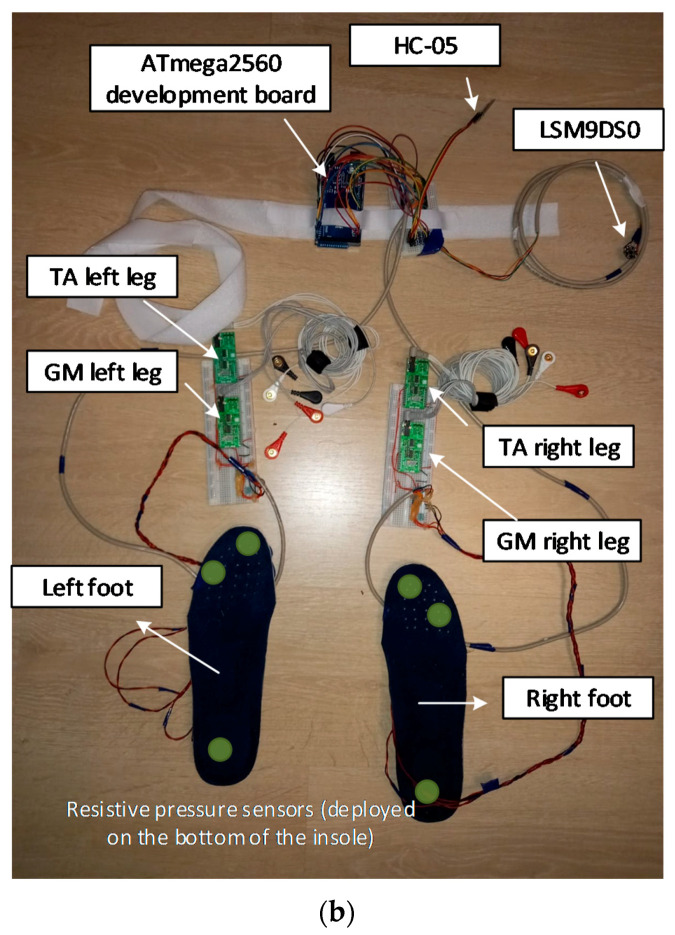
The proposed wearable physiograph for gait monitoring in PD: (**a**) block diagram and (**b**) practical realization.

The proposed gait monitoring physiograph is presented in the block diagram from Figure 1a and the practical realization from Figure 1b.

The proposed wearable physiograph is developed around an ATmega2560 microcontroller (µC), which reads six Aidong IMS C20B thin-film resistive pressure sensors and four EMG channels over the analog ports and a LSM9DS0 module over the I2C interface. Signal acquisition is performed with synchronized sampling, with an fs = 100 Hz sampling frequency and an on-chip 10-bit analog-to-digital converter (ADC). Under this setup, the proposed physiograph performs bilateral tracking of the foot biomechanics through the plantar pressure progression pattern, lower-limb muscular activation, and unilateral monitoring of the arm balance.

**Figure 2:**
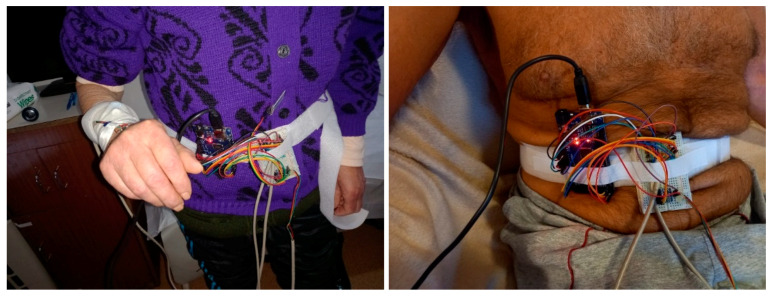
Illustration of the waist-worn ATmega2560 microcontroller development board, attached to a Velcro strip, which reads the peripherals involved in the bilateral monitoring of foot biomechanics and unilateral tracking of the arm balance.

The µC development board is attached to a Velcro strip and is worn around the user’s waist, as illustrated in Figure 2.

Foot biomechanics is assessed using three pressure sensors and two EMG channels, clustered into a foot biomechanics assessment module [43]. Two such modules are considered for bilateral monitoring.

**Figure 3:**
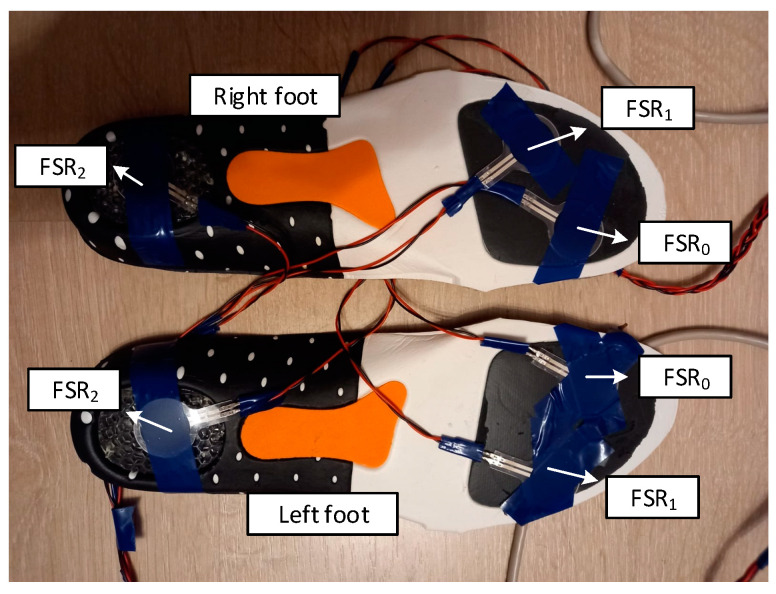
Illustration of the resistive pressure sensor placement onto the shoe insoles, under the toe, metatarsal arch, and heel area, respectively, for bilateral tracking of the plantar pressure progression pattern along the center of pressure progression line.

Operation of the foot biomechanics assessment module is described as follows: bilateral tracking of the plantar pressure progression pattern during the gait cycle is performed using three pressure sensors, attached onto an insole below the toe (FSR_0_), metatarsal arch (FSR_1_), and heel area (FSR_2_), respectively, following the center of pressure (COP) progression line, as illustrated in Figure 3.

**Figure 4:**
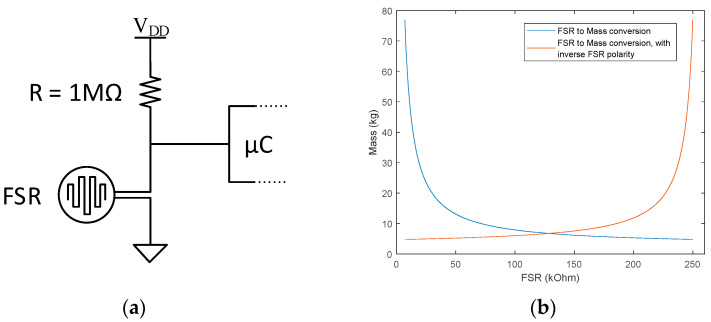
Deployment of the resistive pressure sensors for operation as force sense resistors: (**a**) schematics, (**b**) mass vs. resistance conversion characteristics.

The sensors are deployed into a resistive divider topology with a *R* = 1 MΩ resistance, as illustrated in Figure 4a, and operate as force sense resistors (FSR) with the sensor resistance value derived as

(1) $$FSR=\frac{V_{FSR}}{V_{DD}-V_{FSR}}\cdot R,$$

where *V_DD_* = 5 V is the supply voltage and *V_FSR_* is the FSR voltage drop. The sensor resistance (kΩ) can be converted to mass (kg) according to the mass vs. resistance characteristics provided in the sensor datasheet and plotted in blue in Figure 4b. Mass can further be converted to pressure (kg/cm^2^) by dividing the mass to the sensor area.

In this work, we target gait pattern assessment rather than podiatric assessment. As such, the FSR resistance derived with (1) is sufficient to indicate the application of plantar pressure. In addition to (1), we have changed the polarity of the FSR signal,

(2) FSR=max(FSR)−FSR,

to have the “HIGH” signal level indicating pressure, and the “LOW” signal level indicating absence of pressure. This also changes the mass vs. resistance characteristics as plotted in red in Figure 4b.

**Figure 5:**
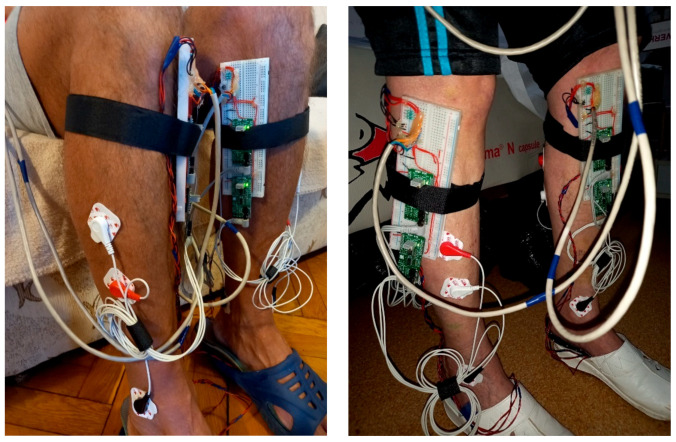
Illustration of the electrode placement for EMG acquisition of the Tibialis anterior and Gastrocnemius medialis, with the reference electrodes placed onto the lateral and medial malleolus, respectively.

Tracking of the lower-limb muscular activation pattern during the gait cycle is performed with two EMG channels which acquire the EMG of the Tibialis anterior (TA) and Gastrocnemius medialis (GM) muscles. Off-the-shelf MikroElektronika EMG Click boards were used for each EMG channel analog front end (AFE), respectively. Wet Ag/AgCl electrodes were employed for EMG acquisition. Electrode placement is illustrated in Figure 5, with the active electrodes (white and red) placed onto the TA and GM muscles and the reference electrodes (black) placed onto the lateral and medial malleolus, respectively.

A 10× AFE gain is set from the on-board potentiometer to accommodate the prescribed 1 µV–10 mV EMG amplitude range, accounting for 2 mV motor unit action potential (MUAP) amplitude of the healthy muscle, 0.5 mV MUAP amplitude for primary muscular disease, as well as 10 mV MUAP amplitude of intramuscular sprouting and chronic partial denervation [43,44,45,46]. On-board filtering is performed with three analog filter stages: two high-pass filters with the cutoff frequencies set to 1.6 Hz and 0.16 Hz, respectively, and a low-pass filter with the cutoff frequency set to 60 Hz.

After acquisition, EMG signal processing accounts for averaging with an 8-sample rectangular window with 50% overlap, a 4th order Butterworth approximation high-pass filter with *f_cL_* = 0.5 Hz to suppress the DC component, and then a 4th order Butterworth approximation low-pass filter with *f_cH_* = 10 Hz. To be noted is that the low-pass frequency of 10 Hz was considered as we were interested in the identification rather than evaluation of muscular activity [47].

**Figure 6:**
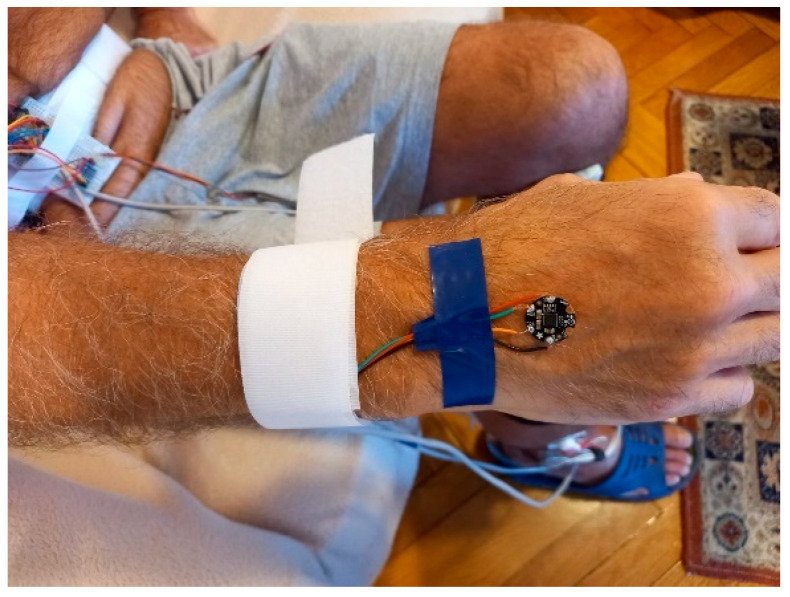
Illustration of the attachment of the LSM9DS0 accelerometer to the user’s right wrist, for arm balance tracking during the gait cycle.

The accelerometer from a LSM9DS0 module is employed to perform arm balance monitoring. The sensor is attached to the patient’s right-hand wrist using a Velcro strip and, if necessary, tightened with an adhesive band, as illustrated in Figure 6.

The accelerometer was configured for a 2G acceleration range, and the sensor data were read using the Adafruit LSM9DS0 library. Accelerometer signal processing assumes averaging with an 8-sample rectangular window with 50% overlap, a 4th order Butterworth approximation high-pass filter with *f_cL_* = 0.5 Hz to suppress the DC component standing for the accelerometer initial position [48], and then a 4th order Butterworth approximation low-pass filter with *f_cH_* = 30 Hz. These filter specifications cover the targeted 1 Hz–10 Hz frequency range of gait-related informational content (most relevant information is available up to 4 Hz) [49], as well as the 4 Hz–6Hz frequency range of tremor [50]. Additionally, the low-pass filter suppresses higher frequency components due to, for example, vibrations as well as noise. The raw signals on the three axes are then converted to acceleration and expressed in m/s^2^.

Two metrics are employed for arm motion tracking based on the dynamic acceleration, defined as follows: the magnitude of acceleration (MA) is determined by applying the Pythagorean theorem to the readings on the three axes, respectively [51], according to equation:

(3) $$MA=\sqrt{x^{2}+y^{2}+z^{2}},$$

and defines the absolute acceleration value. The variation of acceleration (VA) has the average of the past readings subtracted from each axis, respectively [47], as defined by equation:

(4) $$VA\left(k\right)=\sqrt{{\left(x\left(k\right)-x_{avg}\left(k\right)\right)}^{2}+{\left(y\left(k\right)-y_{avg}\left(k\right)\right)}^{2}+{\left(z\left(k\right)-z_{avg}\left(k\right)\right)}^{2}},$$

where *k* is the current index and

(5) $$x_{avg}\left(k\right)=\frac{1}{k-1}\cdot \sum_{i=1}^{k-1}x_{i},$$

(6) $$y_{avg}\left(k\right)=\frac{1}{k-1}\cdot \sum_{i=1}^{k-1}y_{i},$$

(7) $$z_{avg}\left(k\right)=\frac{1}{k-1}\cdot \sum_{i=1}^{k-1}z_{i},$$

are the average of the past readings, i.e., up to index *k −* 1 on each axis, respectively.

**Figure 7:**
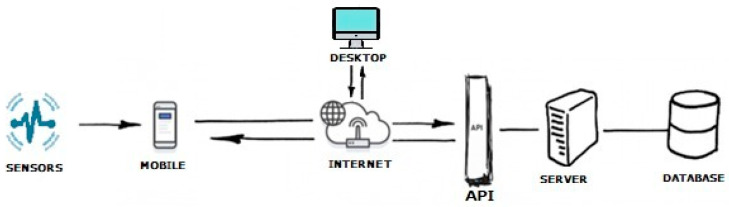
Diagram of the implemented solution to have the signals recorded from the users wearing the gait monitoring physiograph transmitted to a mobile terminal over a Bluetooth radio link and stored into an online database for future retrieval.

The proposed physiograph is aimed at long-term monitoring in a ubiquitous healthcare environment. Wireless connectivity is achieved by deploying the proposed physiograph with a HC-05 Bluetooth module. After acquisition, the raw data are sent over UART to the HC-05 module and transferred to an Android mobile terminal, e.g., a smartphone or tablet. The mobile terminal collects the user data from the physiograph over Bluetooth and assembles it into JavaScript Object Notation (JSON) files. The JSON files are sent one by one over the Internet to the server via REST API and stored in an online database. From this database, the signals are available for later retrieval to a desktop computer. A diagram of the application is presented in Figure 7.

**Figure 8:**
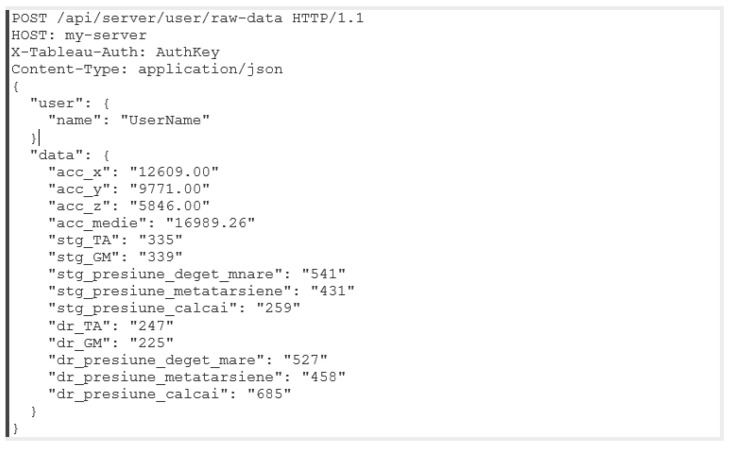
Example of a message sent to the API via the mobile phone.

**Figure 9:**
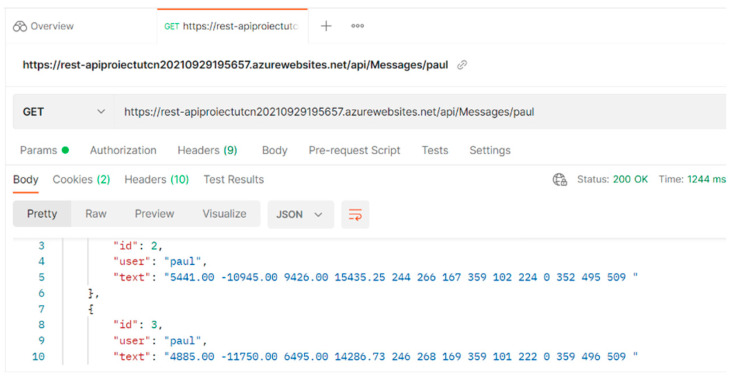
The online database content, resembling the data acquired with gait monitoring physiograph.

An example of a message sent to the API via the mobile phone is provided in Figure 8. The data will be stored in an online database. An example of the database content in Figure 9 illustrates that the data dictionary holds all the data acquired with the gait monitoring physiograph and is made available for later retrieval onto a laptop or personal computer for processing and interpretation.

### 2.2. Gait Assessment—Correlation

**Figure 10:**
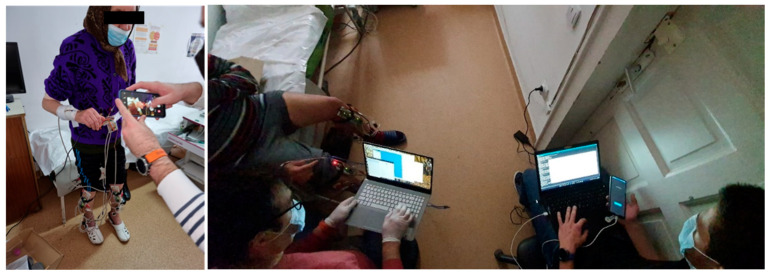
Photographs taken during the trials of gait assessment.

The study group was instructed to undertake steady-state walking, at a pace of their own choice, and walk around the room. One or two walking trials were performed to make sure that the users were comfortable with the wearable devices and that they understood the requirements of the exercise. The subsequent steady-state walking activity was then recorded for the proposed gait monitoring and assessment procedure. Photographs taken during the trials of gait assessment are illustrated in Figure 10.

A physiological gait cycle is considered from one heel strike to the next heel strike of the same foot and consists of a stance and a swing phase, respectively [52,53]. The stance phase is further split into:

1. Heel strike—with pressure applied onto the heel area;
2. Support (or foot flat);
3. Midstance;
4. Heel off—with pressure applied onto the metatarsal arch and hallux;
5. Toe off—with pressure applied only onto the hallux before lift-off [43].

The physiological gait cycle is associated with arm balance, which describes a forward sway during the stance phase (from heel strike to lift-off) and a backwards sway during the swing phase (from lift-off to the next heel strike) [43]. This is visible on the arm balance MA waveform which describes a U-shaped pattern. The MA maxima account for the arm sway direction changes, constituting a good indicator for the stance phase initiation and ending.

The gait pattern in PD differs from the physiological gait. The literature describes a flat-foot strike for the PD gait pattern, or toe-to-heel plantar pressure progression in more advanced stages [54], associated with reduced lifting of the foot after lift-off [55] and limited or no arm balance along the gait cycle [56]. As such, the MA waveform exhibits a larger number of peaks, corresponding to the oscillations of the body’s center of mass and tremor. In this case, the VA waveform exhibits a larger variability corresponding to the increased number of MA peaks.

**Figure 11:**
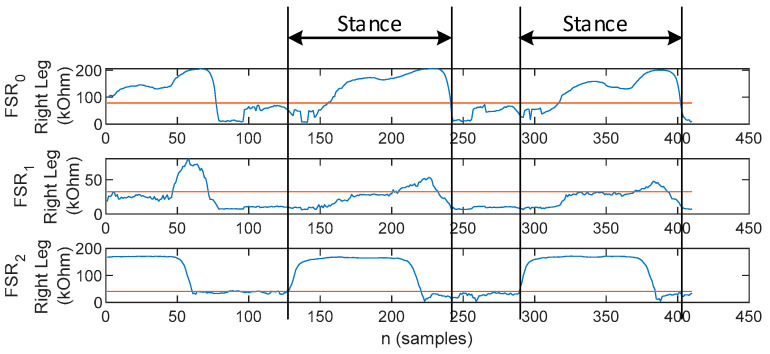
Illustration of the procedure for stance identification: the stance phase lasts from the first to the last occurrence of plantar pressure, identified by having the FSR signals compared to an empirical threshold level.

Due to the large number of peaks, the MA waveform cannot be employed to provide indication regarding stance initiation and ending in PD. In this work, we have rather employed the plantar pressures for stance identification. Plantar pressure detection was performed by comparing the FSR value to an empirical threshold level *FSR_th_* computed as a fraction of the FSR signals. Accordingly, one stance phase ranges from the first to the last occurrence of plantar pressure, regardless of which pressure point, as illustrated in Figure 11. The stance phases identified in this manner account for the signal frames applied for cross-correlation in the gait assessment procedure described further on.

Physiological gait assumes a precisely defined interdependency between arm balance, plantar pressure, and lower-limb muscular activation. We evaluate signal interdependency in the time domain using the cross-correlation function given by

(8) $$R_{sig_{1},sig_{2}}\left(m\right)=\{\begin{array}{c} \overset{N-m+1}{\underset{n=0}{\sum }}sig_{1}\left(n+m\right)\cdot sig_{2}\left(n\right),\;m\geq 0\\ R_{sig_{1},sig_{2}}\left(-m\right),\;m<0 \end{array},$$

where *sig*_1_ and *sig*_2_ are the signal frames being correlated, *m* is the cross-correlation index, and *N* is the frame length [57]. The cross-correlation function defined in (8) provides a measure of the similarity between the two signals *sig*_1_ and *sig*_2_ as a function of *m*. As such, cross-correlation maxima in the origin account for the identification of signal interdependencies. Provided the cross-correlation maxima are situated outside the origin, the index of cross-correlation peaks accounts for the displacement between the signals.

First, we assess whether the arm balance MA peaks, corresponding to arm balance initiation and ending, are synchronous with the stance initiation and ending determined from the plantar pressure progression pattern. This should be the case for physiological gait. Next, we evaluate the cross-correlation functions for:

1. arm balance MA vs. lower-limb muscular activation signals, i.e., TA and GM respectively,
2. lower limb muscular activation signals vs. FSR signals respectively.

As we move forward, we employ the correlation coefficient matrix to quantify the interdependency between either signal pair. A generic correlation coefficient matrix for *M* signals is expressed as:

(9) $$R=\left(\begin{array}{cccc} 1 & \rho \left(sig_{1},sig_{2}\right) & \cdots & \rho \left(sig_{1},sig_{M}\right)\\ \rho \left(sig_{2},sig_{1}\right) & 1 & \cdots & \rho \left(sig_{2},sig_{M}\right)\\ \cdots & \cdots & \cdots & \cdots \\ \rho \left(sig_{M},sig_{1}\right) & \rho \left(sig_{M},sig_{2}\right) & \cdots & 1 \end{array}\right),$$

where

(10) $$\rho \left(sig_{i},sig_{j}\;\right)=\frac{1}{N-1}\sum_{n=1}^{N}\left(\frac{sig_{i}\left(n\right)-\mu_{sig_{i}}}{\sigma_{sig_{i}}}\right)\left(\frac{sig_{j}\left(n\right)-\mu_{sig_{j}}}{\sigma_{sig_{j}}}\right),\;i,j=\bar{1,M},$$

is the Pearson correlation coefficient of two signals *sig_i_* and *sig_j_*; *µ* and *σ* are the mean and standard deviation of the signals indicated in the signal subscripts, respectively; and *N* is the signal length [58,59]. We aim to generate the correlation coefficient matrix for the signal space

(11) $$SIG=[MA,\;VA,TA_{left},GM_{left},FSR_{0,left},FSR_{1,left}FSR_{2,left},\;TA_{right},GM_{right},FSR_{0,right},FSR_{1,right}FSR_{2,right}],$$

consisting of the arm balance MA and VA (determined from the accelerometer signals), lower-limb EMG and plantar pressures. To be noted is that the main diagonal of the cross-correlation matrix consists of unity elements and accounts for the fact that the signals are correlated to themselves.

In contrast to (8), the definition of the correlation coefficients given in (10) does not account for the displacement between the signals, but only provides a quantification for signal similarity. Physiological delays originating from the biomechanical processes involved during gait, e.g., arm balance initiated before or after heel strike, arm balance terminated before or after heel strike, etc., which are determined using (8) as shifting of the cross-correlation peak form the origin, are missed using the correlation coefficients in (10).

To address the displacement of the signals in between one another and visualize them on the correlation coefficient plot, we have generated 10 shifted versions of the signal frame. Consequently, we extended the signal space to 120 signals which are to be correlated, resulting in a 120 × 120 correlation coefficient matrix. A 10 × 10 section from this matrix illustrates the interdependency between either shifted versions of the signals, rather than the signals themselves. Then, the largest coefficient value, accounting for the best similarity, determines the lag between the signals.

### 2.3. Gait Assessment—AI-Based Decisional Support

AI-based decisional support for the identification of PD gait pattern is implemented in this work using convolutional neural networks (CNN). The CNN is a deep learning algorithm that takes an image as input, assigns importance to the features in the image, and can differentiate them from each other. Thus, this type of network has the ability to extract local features based on the convolution operation between the original bidimensional data and certain series of the convolution kernels. The preprocessing required in a CNN is much lower compared to other classification algorithms, and such networks are used in applications for image recognition.

One of the benefits of deep learning is the ability to generalize and to learn massive amounts of data. Good network generalization capacities are obtained by accounting for the relationship between the size of the learning database and the complexity of the network architecture. The higher this ratio, the better the network performance on the test dataset. Furthermore, a big advantage of CNN networks is the weight sharing feature, which reduces the number of trainable network parameters and in turn helps the network to enhance generalization and avoid overfitting.

In this study, we used several architectures such as MobileNet, EfficientNetB0, and Xception. MobileNet is a CNN architecture model used for image classification and mobile vision. The advantage of this network is the very low computing power to apply transfer learning, because the model is based on depthwise separable convolution that has the effect of reducing the calculations and the size of the model. MobileNet uses 3 × 3 depthwise separable convolutions, using 8 to 9 times fewer calculations than standard convolutions with only a small reduction in accuracy. Counting the deep convolutions as separate layers, MobileNet has 28 layers [60]. The EfficientNet model is based on the uniform scaling of the network width, depth, and resolution to improve performance. This network has been extended to a family of deep learning architectures with very good accuracy and efficiency [61]. Xception is built on two main points: depthwise separable convolution, i.e., a depthwise convolution followed by a pointwise convolution, and shortcuts between convolution blocks.

**Figure 12:**
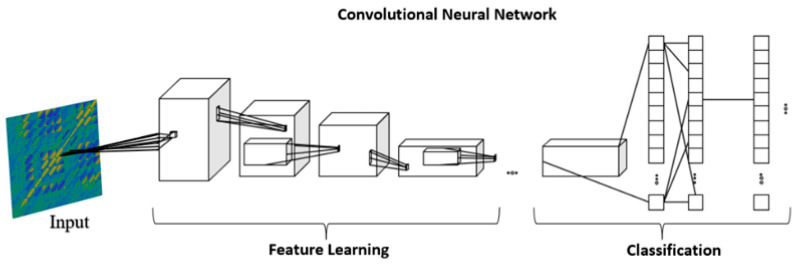
Flowchart of the CNN employed for the classification of the gait pattern into physiological and PD classes.

The surface plot of the correlation coefficient matrix is saved as a jpeg image and is applied to the CNN for classification into physiological and pathological gait. The flowchart of the proposed solution is shown in Figure 12.

The parameters used to train the models are listed in Table 1. A very important parameter is the learning rate, which was chosen to be 0.05 for all models and has the role of controlling the model in response to the estimated error each time the model weights are updated. To reduce the nonlinearity of the output, the Softmax output layer activation function is used for all models. This function determines the type of predictions that the model can make. At the same time, the loss is the prediction error of the network, and the loss function has the role of determining the error. In the proposed binary classification system, the binary cross-entropy compares the predicted probability of the model with the actual result, which can be 0 or 1.

Models were trained on the graphic processing unit (GPU) in Google Colab using Keras. The motivation for Keras is ease of use and extension as neural layers, cost functions, optimizers, initialization schemes, and activation functions. As such, the activation functions are standalone modules that can be combined to create new models defined in Python. Keras offers scalability because it can run on tensor processing units (TPU) or large groups of GPUs, and the model can be exported to run in the browser or on a mobile device.

We have generated a total number of 236 images of correlation coefficient matrix plots, corresponding to the 10-subject database. This constitutes the data set for the CNN which aims to discriminate the walking pattern between physiological gait and PD. Since the data set is small, an augmentation was performed. The Adam optimizer was used to optimize the neural network. The RMSProp optimizer was used in the optimization of the EfficientNetB0 convolutional neural network. Model training was performed with 150 epochs for the MobileNet, EfficientNetB0, and Xception models. The batch size for MobileNet is 32, for EfficientNet it is 64, and for Xception it is 128. To evaluate the performance of each model, the data set was divided as follows: 70% for the training set, 10% for the validation set, and 20% for the test set.

## 3. Results

### 3.1. Gait Assessment—Time Domain

**Figure 13:**
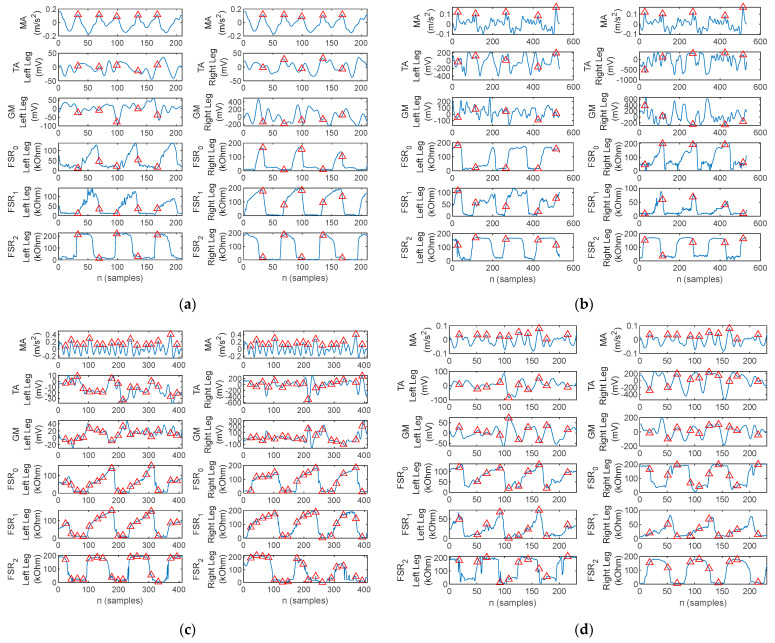
Three-cycle gait section plot of the arm balance MA, bilateral EMG of the TA and GM, and FSR signals, with the red triangles indicating the MA maxima, acquired with the proposed physiograph during walking trials on (**a**) healthy control, (**b**) PD Patient 5, (**c**) PD Patient 1, and (**d**) PD Patient 3.

A section consisting of three gait cycles acquired during the walking trials is illustrated in Figure 13 for one healthy control and three PD patients. The arm balance MA and bilateral EMG of the TA and GM as well as the FSR resistance values, respectively, are plotted. Red markers are placed to indicate the MA maxima.

Figure 13a exhibits the physiological gait pattern (Healthy control 1). Indeed, the peaks of the arm balance MA correspond to the initiation and ending of the stance phases, and the plantar pressures follow the physiological heel→metatarsal arch→hallux progression pattern. Moreover, TA activity can be observed simultaneously with pressure under the heel area and GM activity simultaneously with pressure under the hallux.

In contrast to Figure 13a, the three-cycle gait section plotted in Figure 13b illustrates the gait pattern of a patient with PD (i.e., Patient 5). The walking pattern exhibits bilateral flat-foot strike, which is typical in incipient and mid-stage PD. Muscular activation is present in a direct link to the plantar pressure points. The magnitude of acceleration also exhibits the U-shaped variation due to arm balance, although it is more pronounced during the stance than the swing phase of the gait cycle. However, a series of small-amplitude local peaks originated by tremor are also visible.

The three-cycle gait section recorded on two other patients with PD (i.e., Patient 1 and 3) is plotted in Figure 13c,d. Both patients exhibited flat-foot strike. What stands out, however, on the MA waveform is the absence of arm balance, in which case the peaks are originated by the tremor.

One thing that stands out in Figure 13d for Patient 3 is an asymmetry between the left and right foot during gait. The left foot exhibits a flat-foot strike, typical for PD, although with reduced plantar pressure on the metatarsal arch. The right foot on the other hand exhibits a plantar progression pattern which has toe pressure exerted until the next heel strike with a slight overlap. This accounts for the fact that the patient does not lift the right foot during the sway phase, but rather pulls the right foot with continuous contact between the toe and the ground. According to the FSR plots, the stance phase is terminated when pressure is reduced under the metatarsal arch and tow, i.e., the spike on *FSR*_0_, and the forefoot is only lifted after heel contact.

**Figure 14:**
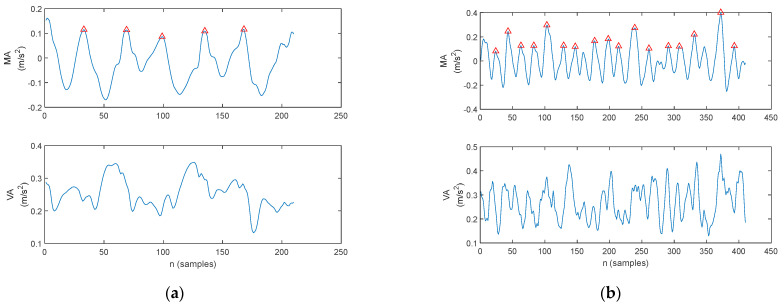
The arm balance MA and VA waveforms during the walking trials, with the red triangles indicating the MA maxima. (**a**) Healthy control—the MA exhibits a U-shaped variation and the VA accounts for smaller variation, and (**b**) PD patient—the MA exhibits a larger number of local peaks with reduced amplitude and the VA accounts for larger variation.

Tremor on the accelerometer signals can be also assessed on the VA waveform. Regarding arm balance, the gait cycle in PD exhibits limited to no arm balance whatsoever. This is clearly visible on the signals acquired from the accelerometers. The arm balance MA of a healthy control, plotted in Figure 14a, exhibits signal peaks (indicated with a red triangle) which correspond to the stance phase initiation and termination, respectively, and a U-shaped waveform corresponding to arm sway. Consequently, the arm balance VA, plotted in Figure 14a, exhibits peaks during the sway.

In contrast, the arm balance MA of a patient with PD exhibits a larger number of peaks, as illustrated in Figure 14b. Although some MA peaks with larger amplitudes, originated by the oscillation of the body center of mass during gait, can be identified as stance initiation and termination (see Figure 13 for a clear identification), most peaks are rather tremor-induced local peaks. As such, the VA waveform reveals a larger variation.

The time-domain plots further enable the assessment of the biomechanical and temporal parameters, used for motor evaluation in accordance with the UPDRS and MDS-UPDRS rating scales for Parkinson’s Disease.

**Figure 15:**
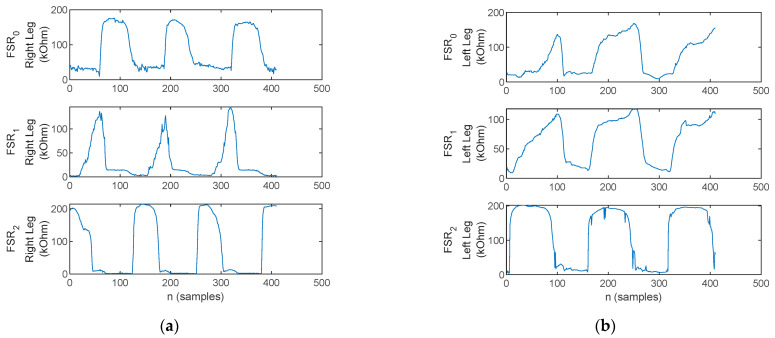
The FSR waveforms (*FSR*_2_—heel, *FSR*_1_—metatarsal arch, *FSR*_0_—toe) during the walking trials for (**a**) healthy control—exhibits the physiological heel→metatarsal arch→toe pressure progression pattern, and (**b**) Patient 5—exhibits flat-foot strike as pressure is applied simultaneously on all three FSRs.

Heel strike accounts for initial contact with the ground at the stance initiation phase under a physiological gait pattern. Heel strike identification is performed by following the plantar pressure progression pattern. The identification of a heel strike is provided by a heel→metatarsal arch→toe pressure progression detection. For illustration, the FSR signals during gait initiation, with heel strike and flat-foot strike, are plotted in Figure 15.

**Figure 16:**
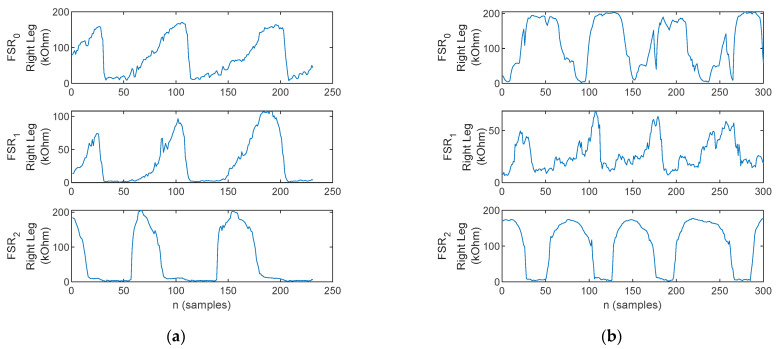
The FSR waveforms (*FSR*_2_—heel, *FSR*_1_—metatarsal arch, *FSR*_0_—toe) during the walking trials for (**a**) healthy control—exhibits a section when no pressure is applied, thus indicating lift-off, and (**b**) Patient 3—exhibits no section when no pressure is applied, indicating that the patient does not lift the foot from the ground but rather pulls the foot during swing.

Lift-off accounts for the fact that the foot is lifted from the ground during the swing phase, and consequently stance phase with the opposite foot. Accordingly, no plantar pressure should be recorded during the swing phase, provided the foot is fully lifted from the ground, accounting for the identification of lift-off. For illustration, the FSR signals during sway, with lift-off and without lift-off, are plotted in Figure 16.

The biomechanical parameters of the test group are listed in Table 2.

The biomechanical parameters of gait, assessed based on the signals recorded with the proposed physiograph, point out the asymmetry between the stance phases with the left and right foot, respectively. The patients exhibit flat-foot strike, which is typical for PD in incipient and middle stages. Only one instance of absent lift-off was observed for Patient 3. Arm balance was identifiable for Patients 1, 4, and 5 who exhibit a small-magnitude tremor.

The healthy control group was not previously diagnosed with any podiatric condition. As illustrated in Table 2, the healthy control group exhibits a physiological gait pattern, except for Healthy Control 2 who exhibits flat-foot strike.

The temporal gait analysis parameters are determined from the time-domain plots of the gait monitoring signals as follows [38,62]:

- Cadence, i.e., the number of steps per minute, is determined as the number of gait cycles in a 10 s segment’s gait signals and extrapolated to one minute.
- Single support, i.e., pressure is exerted by only one foot and not the other, determined from a 10 s segment of the gait signals, and expressed in percentages.
- Double support, i.e., pressure is exerted by both feet during double limb support, determined from a 10 s segment of the gait signals, and expressed in percentages.
- Ratio of the single support to double support (s/d) is the ratio for single support and double support.
- Stride time variability is expressed as a coefficient of variation (CoV) computed from the mean (*µ_stride_*) and standard deviation (*σ_stride_*) of the strides over a 10 s segment of the gait signals [62,63,64], as given in the following equation
  (12) $$CoV=\frac{\sigma_{stride}}{\mu_{stride}}\cdot 100,$$
  and is expressed in percentages.

To be noted is that a 10 s interval is sufficient for the assessment considering that the walking trials assume steady-state walking. The temporal parameters of the test group are listed in Table 3.

What stands out in both Healthy control group and PD group is that the cadence is considerably smaller than the nominal 60 steps/min [52,53]. Both healthy controls and PD patients expressed that their pace was reduced because of physiograph wiring.

The PD group exhibits reduced percentage of single support and S/D in comparison to the healthy control group, while the percentage of double support is not significantly different. On the other hand, stride time variability is increased for the PD group in contrast to the healthy control group.

What stands out for the PD group is that Patient 4 exhibits a very small percentage of single support with the left leg. This is because Patient 4 has a very small right leg lift-off during the swing phase of the gait cycle.

### 3.2. Gait Assessment—Cross-Correlation

As discussed in Section 2, a physiological gait pattern assumes some specifically defined interdependencies between the plantar pressure, lower-limb muscular activation, and arm balance, assessed using the cross-correlation function. Illustration of the cross-correlation between the signals acquired with the proposed physiograph is presented next.

**Figure 17:**
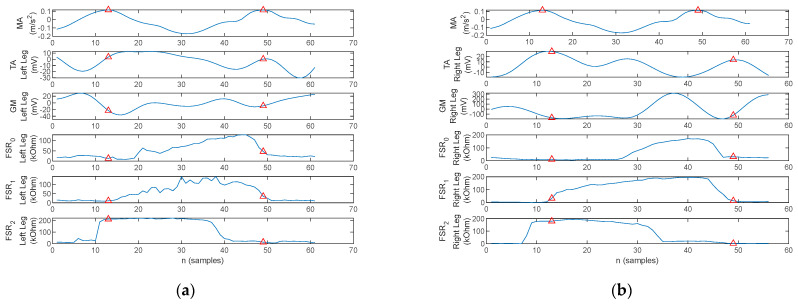
One-stance gait section plot of the arm balance MA, EMG of the TA and GM, and FSR signals for a healthy control, with the red triangles indicating the MA maxima: (**a**) left foot and (**b**) right foot.

The arm balance magnitude of acceleration, the TA and GM activation, and the FSR signals for a stance-phase, acquired on a healthy control for both the left foot and the right foot, are plotted in Figure 17. Red markers indicate stance initiation and termination, respectively.

**Figure 18:**
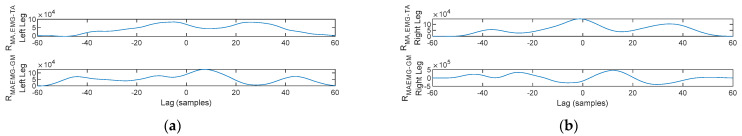
Cross-correlation of the MA and EMG signals plotted in Figure 17 for the (**a**) left foot and (**b**) right foot.

The cross-correlation of the arm balance MA to the lower-limb muscular activation is plotted in Figure 18. As illustrated for the left foot, *R_MA,EMG-TA_* exhibits a peak to the left of the origin, indicating a delay of 70 ms of the TA activation vs. the MA stance initiation peak. Similarly, *R_MA,EMG-GM_* exhibits a peak to the left of the origin, indicating an advancement of 70 ms of the GM activation vs. the MA stance termination peak. Nevertheless, both delays are accounted for as physiological. A similar reasoning can be formulated for the right foot, indicating a delay of 20 ms of the TA vs. the MA stance initiation peak and an advancement of 110 ms of the GM activation vs. the MA stance termination peak.

**Figure 19:**
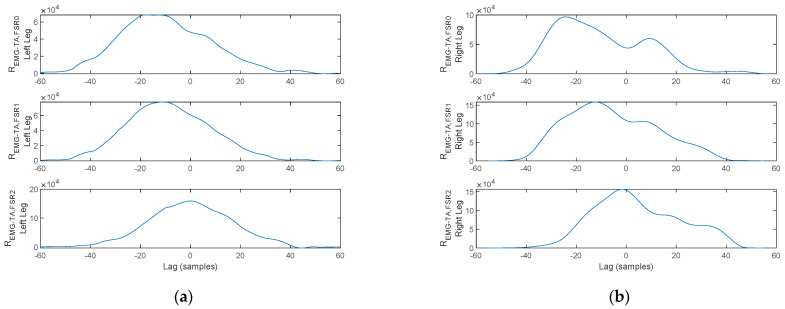
Cross-correlation of the TA and FSR signals plotted in Figure 17 for the (**a**) left foot and (**b**) right foot.

Next, the cross-correlation of the TA activation signal to the FSR signals is plotted in Figure 19. As illustrated, *R_EMG-TA,FSR_*_2_ exhibits a cross-correlation peak in the origin, indicating a direct interdependency between TA contraction and plantar pressure under the heel area. In contrast, the peaks of *R_EMG-TA,FSR_*_1_ and *R_EMG-TA,FSR_*_0_ are shifted from the origin, indicating that plantar pressure under the metatarsal arch and the hallux are not directly linked to the TA. The lag indicates the displacement, i.e., delay, between heel strike (TA contraction), heel off (*FSR*_1_ and *FSR*_0_), and toe off (only *FSR*_0_). This reasoning holds for both feet.

**Figure 20:**
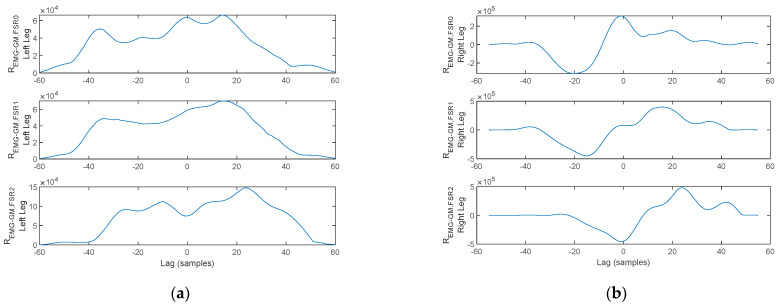
Cross-correlation of the GM and FSR signals plotted in Figure 17 for the (**a**) left foot and (**b**) right foot.

A similar reasoning is formulated regarding the cross-correlation of the GM activation signal to the FSR signals plotted in Figure 20. As illustrated, *R_EMG-TA,FSR_*_0_ exhibits a cross-correlation peak in the origin, indicating a direct interdependency between GM contraction and plantar pressure under the hallux. In contrast, the peaks of *R_EMG-TA,FSR_*_2_ and *R_EMG-TA,FSR_*_1_ are shifted from the origin indicating that plantar pressure under the heel area and metatarsal arch are not directly linked to the TA. The lag indicates the displacement, i.e., delay, between heel strike (*FSR*_2_), heel off (*FSR*_1_ and *FSR*_0_), and toe off (GM activation). This reasoning holds for both feet.

**Figure 21:**
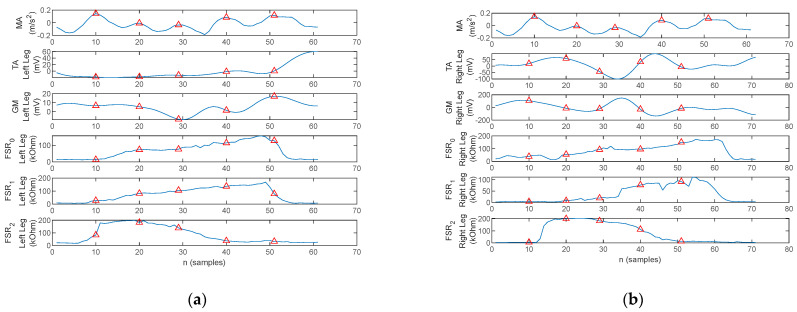
One-stance gait section plot of the arm balance MA, EMG of the TA and GM, and FSR signals for a PD patient, with the red triangles indicating the MA maxima: (**a**) left foot and (**b**) right foot.

In contrast, the arm balance MA, the TA and GM activation, and the plantar pressures for a stance-phase acquired under a bilateral monitoring scenario from a PD patient are plotted in Figure 21.

**Figure 22:**
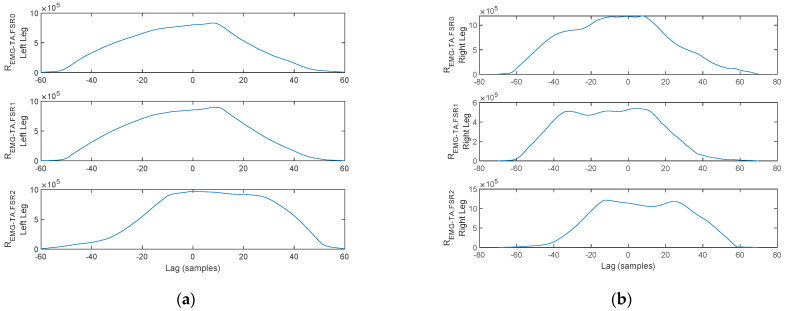
Cross-correlation of the TA and FSR signals plotted in Figure 21 for the (**a**) left foot and (**b**) right foot.

**Figure 23:**
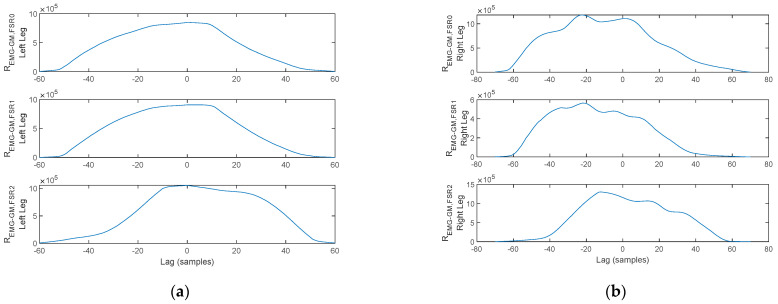
Cross-correlation of the GM and FSR signals plotted in Figure 21 for the (**a**) left foot and (**b**) right foot.

Two aspects stand out in Figure 21. On one hand, the MA peaks are not in a direct correspondence with the onset and termination of stance. With this consideration, the cross-correlation of the MA to either one of the lower-limb muscles becomes irrelevant, as the cross-correlation peaks indicate correspondence of the muscular activation to MA maxima originating from tremor rather than arm balance. On the other hand, the gait pattern exhibits flat foot strike for the left leg, identified by having pressure exerted simultaneously by the heel, metatarsal arch, and hallux, respectively, rather than the physiological heel→metatarsal arch→toe progression. This latter remark results in cross-correlation maxima in the origin, or close to the origin, for all signal pairs, as plotted in Figure 22 and Figure 23.

**Figure 24:**
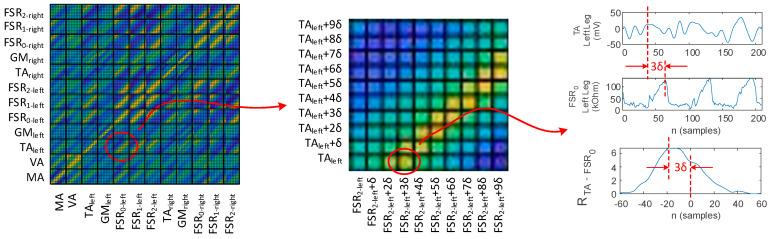
The surface plot of a correlation coefficient matrix split in 12 × 12 sections corresponding to the monitored signal pairs, with a 10 × 10 section providing the quantification of interdependency on shifted versions of the corresponding signals.

The surface plot of the correlation coefficient matrix defined in (9) was employed in this work to visualize the biomechanical and temporal parameters of gait. Consider, for exemplification, the surface plot of the correlation coefficient matrix illustrated in Figure 24. Each 10 × 10 matrix section, delimited by the squares, displays 100 correlation coefficients for the shifted versions of the signal pairs indicated in the matrix header. Yellow corresponds to large coefficient values, i.e., good similarity between the signals, whereas blue corresponds to small coefficient values. As such, the largest correlation coefficient values, i.e., the brightest colors in the correlation coefficient matrix surface plot, yield the displacement between the signals given in the matrix header. As shown, the displacement determined from the correlation coefficient matrix is equal to the one determined using the cross-correlation function in Figure 19.

**Figure 25:**
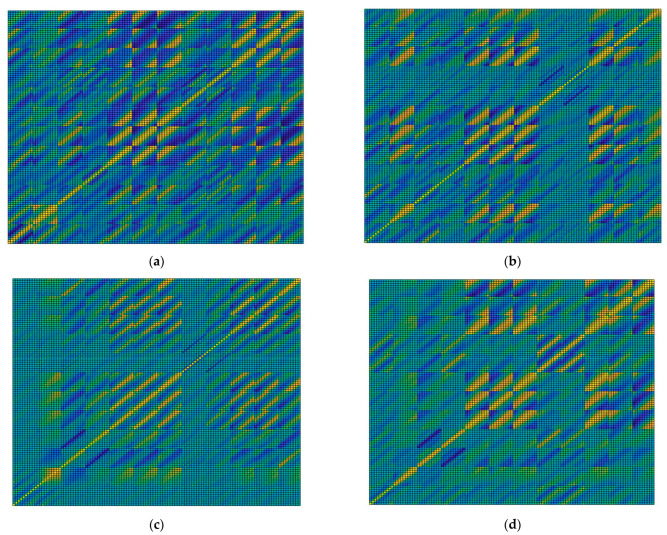
The correlation coefficient matrix surface plots, generated from the signals acquired with the proposed physiograph during the walking trials, for (**a**) healthy control, (**b**) Patient 5, (**c**) Patient 1, and (**d**) Patient 3.

For illustration, the surface plot of the correlation coefficient matrix computed for the healthy control and the three PD patients from Figure 13 is illustrated in Figure 25. Interpretation of the correlation coefficient matrices follows in Section 4—Discussion.

### 3.3. Gait Assessment—AI-Based Decisional Support

AI-based decisional support for the discrimination of the PD-specific gait pattern was implemented using CNNs. The CNN takes the jpeg of the correlation coefficient matrix surface plot to classify the gait into physiological or PD pattern.

The CNN model performances were evaluated based on accuracy (Acc), sensitivity (Se), specificity (Sp), and precision, i.e., positive predicted values (PPV). These are defined using the true positive (TP), false positive (FP), true negative (TN), and false negative (FN) counts, respectively, as indicated in Equations (13)–(16). TP and TN count the number of correct classifications of Parkinsonian and physiological walking, respectively, whereas FP and FN count the number of incorrect classifications. According to Equations (13)–(16), Acc shows the probability of correct classification, Se evaluates the model’s ability to identify the true positive samples, Sp evaluates the model’s ability to identify the true negative samples, and PPV indicates the probability of the model to correctly classify a sample as positive. Finally, the error is then computed as the difference between the model training result and the desired result.

(13) $$Acc=\frac{TN+TP}{TP+TN+FP+FN},$$

(14) $$Se=\frac{TP}{TP+FN},\;$$

(15) $$Sp=\frac{TN}{TN+FP},$$

(16) $$PPV=\frac{TP}{TP+FP}$$

At the end of the training and data validation process, the performance metrics were calculated and listed in Table 4, suggesting that this algorithm for training a CNN network achieves efficient identification. As indicated, the best results were obtained on the MobileNet model with 95% accuracy, 90% sensitivity, and 95% precision. The best results were obtained on the MobileNet model.

The best performance metrics which we achieved in this work are listed in Table 5 for further comparison with the classification performances achieved by some classifiers reported in the literature, including Random Forest and Support Vector Machines (SVM).

## 4. Discussion

The work described in this article is developed in the context of the PDxOne research project, which aims to implement AI-based support for the collection and interpretation of medical data in PD, in the framework of ubiquitous healthcare.

The application described in this paper targets gait monitoring and assessment, based on the foot biomechanics, i.e., plantar pressure and lower-limb EMG, in correlation to upper-limb balance. To evaluate the gait problems that characterize PD, clinicians use semiquantitative rating scales such as the unified Parkinson’s disease rating scale (UPDRS) [66] or the movement disorders society unified Parkinson’s disease rating scale (MDS-UPDRS) [42]. Objective gait evaluation was performed in this work accordingly. For qualitative gait assessment, we have evaluated biomechanical parameters expressed in terms of arm balance, heel strike, and foot lift-off, with the results listed in Table 2. For quantitative gait assessment, we have evaluated temporal parameters expressed in terms of cadence, single support, double support, single support to double support ratio, and stride time variability, with the results listed in Table 3.

Gait impairment is evolving throughout the progression of the disease, and the patterns of gait disturbances that are detected can differ from early to mild/moderate and advanced stages of PD [67], but the relationship between gait features and disease progression is not completely explained [68].

In PD, non-motor symptoms such as anxiety, depression, and cognitive impairment develop along with the motor symptoms, influencing the subjects’ ability to perform motor tasks [9]. In the presence of such non-motor symptoms, motor tasks performed under trial conditions with a device attached to the body become a real challenge to the patient. Indeed, having the physiograph modules attached to the body produces an unusual sensorial stimulation to the patient. On the other hand, the presentation to the doctor’s office or the medical laboratory is a stress factor itself for many patients, which strikes the emotional component. Consequently, we expected the gait analysis results of the study group to be influenced: smaller stride length, slower gait velocity, and smaller activity motor unit recruitment, although gait is an activity of daily living. Indeed, the results reported in Table 3 illustrate that both PD patients and healthy controls exhibit a smaller cadence compared to the nominal 60 steps/min [43,52,53]. As a natural consequence of the reduced cadence, Table 3 also shows longer single-support and shorter double-support durations compared to the nominal 30% of the gait cycle [43,52,53].

Gait physiology was further presented in a visual manner using the correlation coefficient matrix defined in Section 2.2. Identification of the gait pattern on the correlation coefficient matrix is discussed as follows: physiological gait, as monitored with the proposed physiograph and illustrated in the correlation coefficient matrix surface plot from Figure 25a, accounts for a heel→metatarsal arch→hallux plantar pressure progression pattern in direct correlation to the lower-limb muscular activation. The plantar pressure progression pattern is visualized in the plantar pressure correlation sections by the shift of the in-section yellow diagonal toward the matrix main diagonal. Shifting of the in-section diagonal away from the matrix main diagonal would account for an inverse plantar pressure progression pattern, namely hallux→metatarsal arch→heel.

Activation of the TA accounts for eccentric contraction during heel strike and initial double limb support [43,52,69], which is visualized by the yellow diagonal in the TA-FSR correlation sections, for both left and right feet, respectively. Next, activation of the TA accounts for concentric contraction during the swing phase [43,52,69], which is visualized by the yellow areas in the top left and bottom right, i.e., dark blue diagonal, in the TA-FSR correlation sections for opposing feet.

Activation of the GM describes eccentric contraction during midstance and concentric contraction during heel off and toe off [43,52,69], which is visualized in the GM-FSR correlation sections for both left and right foot, respectively. The GM is inactive during the swing phase, which is visualized in the GM-FSR correlation sections for opposing feet.

Physiological gait assumes a complete arm balance during the gait cycle, with the forward swing accounting for right foot stance and the backward swing accounting for left foot stance. As such, the arm balance is fully correlated to the lower-limb EMG and plantar pressure signals, as visualized in the MA-EMG and MA-FSR correlation sections. To be noted is that only the right arm was considered for assessment in the present work, which explains the yellow diagonal in the correlation sections of the MA with the opposite, i.e., left foot, and the yellow corner areas (dark blue diagonal) in the correlation sections of the MA with the same side, i.e., right foot. Indeed, the forward swing of the right arm−left foot stance produces a larger magnitude of the U-shaped MA signal, in contrast to the backward arm swing−right foot stance, which produces a smaller magnitude (see Figure 14). The VA signal on the other hand exhibits peaks during midstance, thus being correlated to the MA, which explains the yellow diagonal on the MA-VA correlation section. Furthermore, the VA is correlated to shifted versions of the EMG and FSR signals, which explains the yellow areas in the VA-EMG and VA-FSR correlation sections.

Parkinsonian gait is clearly distinguishable from the physiological gait. One of the most representative but non-specific early features of Parkinsonian gait is reduced speed [67]. It has been demonstrated that early PD subjects exhibit a reduced amplitude of arm swing and smoothness of locomotion, as well as increased interlimb asymmetry, all of these being more specific to PD and often the first motor symptoms [70].

Such features of the Parkinsonian gait pattern are identifiable on the correlation coefficient matrix. Some gait features attributable to PD, which we were able to identify and assess during the clinical test of the proposed physiograph, are presented as follows: the correlation coefficient matrix surface plot from Figure 25b corresponds to Patient 5, whose walking pattern described in Table 2 consists of flat-foot strike, bilateral lift-off, presence of arm balance, and small tremor. In contrast to the healthy control, the flat-foot strike is visualized in the left-foot FSR−FSR correlation sections as a yellow main diagonal. Plantar pressure is applied simultaneously to all sensors during flat foot strike, and consequently the FSR signals are correlated to one another (see Figure 13b). The patient keeps a regular lower-limb muscular response during the stance phases, visualized by the TA-FSR and GM-FSR correlation sections. The arm balance is also present, visualized by the MA-EMG and MA-FSR correlation sections. Tremor, although existent, is small in magnitude and consequently allows for the visualization of the yellow diagonals in the VA-EMG and VA-FSR correlation sections.

The correlation coefficient matrix surface plot from Figure 25c corresponds to Patient 1, whose walking pattern described in Table 2 exhibits bilateral flat-foot strike, bilateral lift-off, presence of arm balance, and large tremor. The correlation coefficient matrix follows the same pattern regarding foot biomechanics as for Patient 5. Regarding tremor, however, Figure 13c shows that the tremor and balance magnitudes in the MA and VA signals are comparable. Consequently, both MA and VA signals are uncorrelated to the EMG and FSR signals, respectively. This is visualized by the correlation sections of both MA and VA to the other monitoring sections, which exhibit a rather uniform coloring.

A different walking pattern was identified for Patient 3, with the correlation coefficient matrix surface plot from Figure 25d, who according to Table 2 exhibits absent heel strike and absent lift-off for the right foot. In this regard, the patient pulls the right foot during the swing phase of the gait cycle. This is visualized on the correlation coefficient plot by the right-foot FSR−FSR correlation sections and the left FSR−right FSR correlation sections, which deviate from the yellow diagonal pattern.

As the disease progresses bilaterally, the asymmetry might decrease, and movement becomes more bradykinetic [68,71]. At the same time, along with the neurodegeneration, the movement of the limbs becomes more impaired, and the patients develop shuffling steps with the increased need of double-limb support [72,73]. The further decline in gait is also caused by the postural changes, which are altering the kinematics of the gait, as is the case of the stooped posture [74].

The changes in gait worsen and motor fluctuations, dyskinesias, and freezing of gait become frequent and are accompanied by reduced balance and postural control, all of these exposing the patient to a severe risk of falling. More than that, the decline in the motor capacity can lead, in advanced stages, to the need of wheelchair use [75,76,77]. We expect to see these changes in the correlation coefficient matrix to facilitate gait pattern interpretation. This could be a game changer for stage-related personalized medicine therapy options.

The proposed physiograph is also envisioned as a portable monitoring solution. For this purpose, the recorded signals are transmitted over a Bluetooth radio link to a mobile device, e.g., smartphone or tablet, and stored in an online database for future retrieval. As such, the proposed solution is applicable for gait monitoring in PD outside the hospital environment. In this scenario, the monitoring protocol can be extended to include the assessment and quantification of influences exercised by daily activities—both domestic and in the community [36], environmental demands [78], environmental manipulation [37], and dual/multiple tasking [36,37].

The proposed physiograph is in a proof-of-concept phase, which was validated in clinical environment. As such, it was to be expected that the performance of both patients and healthy controls was influenced as reported, due to their subjective perception on the worn-in discomfort. There are two reasons for this. Firstly, the proposed physiograph is still in proof-of-concept phase. In accordance with this, the subjects complained of worn-in discomfort and discomfort from wires interfering with their mobility. Secondly, the gait measurements were conducted in a relatively small room, which likely inhibited the automaticity of walking at a normal pace [79]. The clinical environment created conditions for directed attention during the walking trial. As such, the subjects are aware of the motor task and concentrate on performing the task, which may have led to the abovementioned deviations from standard.

The solution to overcome such issues is the repetition of the motor task for a prolonged period (several days) as well as removing the patient from the clinical environment and placing them into their usual living environment, both inside and outside the home. This is enabled by the wearability and portability features of the proposed physiograph.

In continuation of our research, we aim to optimize the system design toward miniaturization, targeting a wireless body area network topology. One step further, the external environment brings multiple sensory information that can divert the patient’s attention from the motor task. Thus, monitoring in an external environment provides multiple functional pieces of information with high value in the selection of the therapeutic and recovery interventions.

The solution proposed in this article was validated in a clinical environment for specific applicability in PD. Nevertheless, we envision that it can be easily extrapolated to further neurodegenerative conditions with gait affection.

On the other hand, PD does not exclude the presence of age-specific concurrent diseases such as (degenerative) osteoarthritis and/or cardiovascular diseases and their sequelae; comorbidities are common among patients with PD [80]. For example, one of the study group patients (Patient 3) also presents post-AVC hemiplegia with mild upper arm sequelae. In addition, inherent age-induced changes which affect joint function, e.g., sarcopenia, loss of proprioception and balance, and increased joint laxity, cannot be neglected [80]. All are associated with loss of independence and an increased incidence of falls.

Along with the clinical, biological, and imaging exam, the functional exam is also very important (including the functional exam performed with our device) for diagnostic purposes, as well as for monitoring the pharmacological disease management and rehabilitation outcome. It can be also used to guide kinetic trainings, to impose gait rhythms and velocities, and even to aid in dual tasks.

Along with the paraclinical examinations, the clinical testing, including functional performance, is key for diagnostic purposes. Part of this examination could be performed with our device and could be useful to provide guidance in kinetic trainings, to impose gait rhythms and velocities, and to aid in dual tasks. With further improvements, the pharmacological monitoring, disease management, and rehabilitation outcomes are in reach.

## 5. Conclusions

PD clearly demonstrates the complexity of the human body and the difficulty in choosing the appropriate treatment. In this paper, we focused on one of the main motor symptoms in PD, the gait. To develop a foundation for sustainable decision-support medical devices, we aim to include other important motor symptoms, as well as the non-motor ones, in future studies. Non-motor symptoms (e.g., cognitive impairment, depression, anxiety, sleep disorders, pain, and other autonomic disturbances) correlate with advanced age and disease severity [81], so they can be considered suggestive for the prognosis of disease progression. Some of these non-motor symptoms may appear much earlier in the course of disease progression [12]. AI-based assessment of these data could raise the importance of new technologies in prediagnosis and digital monitoring of patients. By pre-diagnostic technologies, we refer to those technologies that have the probabilistic ability to provide data interpretation at a high level of certitude for a given diagnosis, often before it is identified by a specialist without computational aid. In our experience, an additional interesting feature of AI systems is that they can identify non-specific symptoms, provided enough data and solid input are available. Regardless of the future development and promising results, the final diagnosis should be reserved, in our opinion, exclusively for the human medical practitioner, following established ethical/legal rules and evidence-based practice.

Back in 2017 when the PDxOne started, the main background idea was to find solutions for PD management optimization by bridging the gap between medicine and computational engineering. We used this approach in our study. Taking into consideration everything mentioned above, we see the necessity for an increased commitment in multidisciplinary treatment by interdisciplinary teams. In our opinion, this interdisciplinary team should include, at least, the following specialists in addition to the neurologist: general practitioner and dentist, geriatrician, trained medical caregiver for patients with PD, speech therapist, occupational therapist, nutritionist, psychologist/psychotherapist ideally specialized in patients with PD, and pharmacologists. To the abovementioned list, we would add new kinds of professionals that have medical degrees such as in a biomedical field (e.g., bioengineer, bioprinting expert, etc.) combined with knowledge of AI and augmented intelligence, as well as lifestyle strategists (advise patients with their health data), telemedicine, and/or health data analyst/biostatistician. Considering that by 2030 we will lack over 18 million healthcare workers worldwide [82], these future professions will be an asset in bridging interdisciplinary activities, they will provide sustainability in healthcare, and they could serve as good choice for internationally trained medical professionals toward alternative career pathways [83] because of labor migration in the era of globalization. We see today this new professional category in its early phase, with clinics having incorporated bioengineers, deep learning developers, bioprinting experts, and so on on their teams. New research projects are already hard to imagine without the congregation of such a team. As with most advancements, this one will also be led by necessity. In the time of Big Data, such an interdisciplinary team could provide personalized treatment for hospitalized patients and those at home. Perhaps the most important aspect of this interdisciplinary team is the medical data, and how interdisciplinary teams could correlate assessments to identify common patterns for optimizing patient-specific treatments. A good line of action would be the development of an international research platform, with standardized parameters, for automated or semiautomated data input. The assessment algorithms for this platform would benefit from the interdisciplinary approach and the fact that coding and treatment could be performed “at the patient’s bed”. A possible output would be an evidence-based decision support software for optimal dosing between drug and non-drug treatments. With such an effort, adequate treatment could be provided for this complex and multi-faceted disease, as PD is scripted [18]. Such an approach, once established, could offer individualized treatment plans and monitoring programs that would further improve PD management, and with it, socio-economic implications. The ultimate goal of our research community should be to offer affordable precision medicine for everyone.

## Figures and Tables

**Table 1:** CNN training parameters.

CNN Parameters	MobileNet	EfficientNetB0	Xception
Image dimension	160 × 160 × 3	160 × 160 × 3	160 × 160 × 3
Learning rate	0.05	0.05	0.05
Epochs	150	150	150
Batch size	32	64	128
Optimizer	Adam	Adam	Adam
Loss function	Binary Cross Entropy	Binary Cross Entropy	Binary Cross Entropy
Output layer activation function	Softmax	Softmax	Softmax

**Table 2:** Biomechanical parameters of the test group.

Test Group	Arm Balance	Tremor	Heel Strike	Lift-Off
Healthy control 1	Present	Absent	Left: present	Left: present
			Right: present	Right: present
Healthy control 2	Present	Absent	Left: absent (flat-foot strike)	Left: present
			Right: absent (flat-foot strike)	Right: present
Healthy control 3	Present	Absent	Left: present	Left: present
			Right: present	Right: present
Healthy control 4	Present	Absent	Left: present	Left: present
			Right: present	Right: present
Healthy control 5	Present	Absent	Left: present	Left: present
			Right: present	Right: present
Patient 1	Present	Large tremor	Left: absent (flat-foot strike)	Left: present
			Right: absent (flat-foot strike)	Right: present
Patient 2	Absent	Large tremor	Left: absent (flat-foot strike)	Left: present
			Right: present	Right: present
Patient 3	Absent	Large tremor	Left: absent (flat-foot strike)	Left: present
			Right: absent	Right: absent
Patient 4	Present	Small tremor	Left: present	Left: present
			Right: present	Right: present
Patient 5	Present	Small tremor	Left: absent (flat-foot strike)	Left: present
			Right: present	Right: present

**Table 3:** Temporal parameters of the test group.

	Cadence (Steps/min)	Single Support (%)	Double Support (%)	S/D	Stride Time Variability (%)
Healthy Control 1	62	Left: 41.8	14.9	Left: 2.8	3.8
		Right: 43.3		Right: 2.9	
Healthy Control 2	27	Left: 36.2	29.8	Left: 1.2	9.8
		Right: 34		Right: 1.1	
Healthy Control 3	24	Left: 35.9	23.4	Left: 1.5	4.8
		Right: 40.8		Right: 1.7	
Healthy Control 4	56	Left: 38.5	17.1	Left: 2.2	3.1
		Right: 44.3		Right: 2.6	
Healthy Control 5	55	Left: 39	19.7	Left: 2	5.7
		Right: 41		Right: 2.1	
Statistics for healthy control group	44.8 ± 17.9	Left: 38.3 ± 2.4	21 ± 5.9	Left: 1.9 ± 0.6	5.4 ± 2.6
		Right: 40.7 ± 4		Right: 2.1 ± 0.7	
Patient 1	39	Left: 38.4	25.6	Left: 1.5	6.3
		Right: 36		Right: 1.4	
Patient 2	44	Left: 29.1	31.6	Left: 0.9	3.4
		Right: 39.2		Right: 1.2	
Patient 3	48	Left: 47	20.6	Left: 2.3	9
		Right: 32.3		Right: 1.6	
Patient 4	32	Left: 48.5	8.9	Left: 5.4	13.1
		Right: 42.5		Right: 4.8	
Patient 5	24	Left: 35	37.5	Left: 0.9	8.2
		Right: 27.4		Right: 0.7	
Statistics for PD group	37.4 ± 9.6	Left: 39.6 ± 8.1	24.9 ± 10.9	Left: 2.2 ± 1.9	8 ± 3.6
		Right: 35.5 ± 5.9		Right: 1.9 ± 1.6	

**Table 4:** Performance metrics of the neural network for gait pattern discrimination.

Performance Metrics	CNN Model		
	MobilNet	EfficientNetB0	Xception
Accuracy	0.95	0.85	0.85
Error	0.19	0.39	0.25
Sensitivity	0.90	0.85	0.9
Specificity	0.96	0.80	0.73
Precision	0.95	0.85	0.74

**Table 5:** Classification performances of the proposed work in comparison to other solutions reported in the literature.

Performance Metrics	Classifier				
	This Work	[32]	[33]	[34]	[65]
Classifier	CNN MobilNet	Random Forest	CNN	CNN	SVM
Accuracy	0.95	0.96	0.63	0.856	0.917
Error	0.19	-	-	-	-
Sensitivity	0.90	0.982	-	0.88	0.752
Specificity	0.96	0.96	-	0.84	0.99
Precision	0.95	0.972	-	0.86	-
